# Two decades of demography reveals that seed and seedling transitions limit population persistence in a translocated shrub

**DOI:** 10.1093/aob/mcu082

**Published:** 2014-05-20

**Authors:** C. L. Gross, D. Mackay

**Affiliations:** Ecosystem Management, University of New England, Armidale, NSW 2351, Australia

**Keywords:** *Olearia flocktoniae*, Asteraceae, Dorrigo daisy bush, extinction patterns, survivorship, life-history analysis, pioneer species, seed bank recovery, endangered species, habitat shift, displaced species, elasticity analysis, sensitivity analyses, local extinction

## Abstract

**Background and Aims:**

*Olearia flocktoniae* is an endangered shrub that was passively translocated from its natural ecosystem, where it has since gone extinct. This study aimed to determine sensitivities vital to populations persisting in human-created areas.

**Methods:**

Population colonization, longevity and extinction were investigated over 20 years using 133 populations. Seed-bank longevity was determined from germination trials of seeds exhumed from extinct and extant sites via a 10-year glasshouse trial and by *in situ* sowing experiments*.* From 27 populations, 98 cohorts were followed and matrix models of transitions from seeds to adults were used to evaluate the intrinsic rate of population growth against disturbance histories. Ten populations (38 cohorts) with different disturbance histories were used to evaluate sensitivities in vital rates.

**Key Results:**

Most populations had few individuals (∼30) and were transient (<5 years above ground). The intrinsic population growth rate was rarely >1 and all but two populations were extinct at year 20. Seeds were short-lived *in situ*. Although >1000 seeds per plant were produced annually in most populations, sensitivity analysis showed that the transition to the seed bank and the transition from the seed bank to seedlings are key vulnerabilities in the life-cycle.

**Conclusions:**

Seedling establishment is promoted by recent disturbance. Increasing the number of disturbance events in populations, even severe disturbances that almost extirpate populations, significantly increases longer-term population persistence. Only populations that were disturbed annually survived the full 20 years of the study. The results show that translocated populations of *O. flocktoniae* will fail to persist without active management.

## INTRODUCTION

Rare species with very restricted distributions are at the forefront of extinction risk simply because all of their populations may experience the same catastrophic event, such as habitat loss. Pioneer species are likely to survive a catastrophe if new opportunities for range expansion occur, which can then be exploited with their r-selected characters. Survivorship of rare species in new locations, though, will depend on overcoming the limitations to population growth and expansion that occurred in the home range. *Olearia flocktoniae* from eastern Australia is a pioneer and rare species of shrub, listed as endangered and not found in naturally disturbed areas since 1916 but only in human-made habitats. This has shifted its range ∼35 km westward from the escarpment of the Great Dividing Range to disturbed areas associated with timber extraction (e.g. plantations and forestry road verges). Little is known about the ecology and persistence of rare species that have been translocated into new habitats. Understanding range shifts in rare species has the potential to inform conservation biology; e.g. exposing weaknesses in the life cycle (Simpson *et al.*, 2005) could reveal where to maximize effort for population persistence in actively translocated endangered species. Therefore, critical evaluation of colonization, persistence and extinction in these species is an important pursuit (e.g. Kammer *et al.*, 2007; Morin and Lechowicz, 2008) and predictive frameworks are required for the many species and communities subject to shifting environmental conditions as a result of rapidly changing climates (Crumpacker *et al.*, 2001; Kaufmann, 2002; Maschinski *et al.*, 2006; February *et al.*, 2007).

Pioneer species offer much for the study of extinction. Their populations come and go repeatedly in measurable time periods and they lend themselves to scrutiny at various scales as their populations exist in confined and temporary habitats (e.g. edge habitats), often over broad geographical areas (spatial scales) and with temporal and spatial differences in the expression of overt (above ground) and covert (seed bank) populations. Incorporation of temporal or spatial scale in these studies enhances our ability to detect whether there are compounding causes of decline. The persistence of a plant species at a place over time is often a function of individual longevity, seed production, seed banking and responses to disturbances, such as herbivory, competition and fire [e.g. *Bertya ingramii* (Scott and Gross, 2004); *Aeschynomene virginica* (Griffith and Forseth, 2005); *Grevillea beadleana* (Smith and Gross, 2002)], yet extinction is a certainty for every population (and species) at some point along a time continuum (Sjöström and Gross, 2006).

Over 20 years, we studied the *de novae* populations of *Olearia flocktoniae* (Dorrigo daisy) in plantations, log dumps and other disturbed areas throughout its range north and west of Dorrigo, NSW, Australia. The aim of our study was to determine which aspects of the species' life history enabled it to persist at some locations and not others. Beckerman *et al.* (2002) make the point that it is necessary to assess the ways in which ‘history in the life-history’ of individuals might influence population dynamics (see also Ehrlen, 2000); here we move up a scale to assess the history of the population (legacy conditions) as an influence on future population longevity. The objectives of the study were to: (1) characterize the parameters associated with population extinction (age of the populations when they go extinct and the stage structure in the final year); (2) compare short-lived populations with longer-lived ones to determine whether a relationship exists between founder population size or total population size and population persistence; (3) determine whether the history of the population size (lifetime size and size in final year before extinction) prior to extinction influences the population size and persistence at re-established sites; and (4) use fecundities and survivorship measures from seed bank to adulthood to build a matrix population model for the determination of sensitivities and elasticities in population growth. We use this information to provide recommendations for management of the species.

## METHODS

### Study location, species and life cycle

*Olearia flocktoniae* (Asteraceae)*,* a spindly shrub, is sparsely distributed as small populations (usually <100 plants) on the Dorrigo Plateau (30°20′24·15″ S, 152°42′46·90″E) in eastern NSW, Australia. The species was discovered in 1909 in a clearing in ‘virgin forest’ near Dorrigo, northern NSW, Australia (Maiden and Betche, 1909). It was collected a few more times in this local area until 1916 and was then presumed extinct until its rediscovery in 1984 on a road verge near Coopernook creek (Murray, 1996), ∼12 km north-east of Dorrigo. The species is a pioneer shrub of forest margins and in its new range these margins are disturbed by road works for forestry activities. The species has not been collected along natural forest edges since 1916. This landscape has been modified for agriculture and the loose scree slopes of the Great Dividing Range where the species most likely existed are now heavily infested with woody weeds such as lantana (*Lantana camara*) and small-leaved privet (*Ligustrum sinense*), which have stabilized these slopes. *Olearia flocktoniae* now exists only in artificially disturbed sites in active forestry areas or areas recently transferred from active forestry to conservation reserves. *Olearia flocktoniae* has been afforded legal protection as a State- and Commonwealth-listed endangered species owing to its restricted range and lack of persistence of populations.

**Fig. 1.**
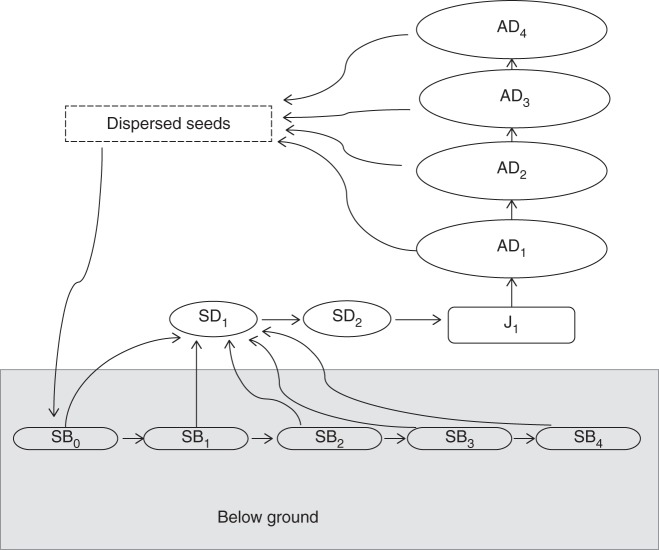
The 12 life cycle stages of *Olearia flocktoniae*: SB_0_, seed bank in the first year at time 0; SB_1_ etc., seed bank in the second year at time 1, etc; SD_1_, germinant <30 d old; SD_2_, seedling >30 d old and <20 cm tall; J_1_, vegetative juvenile; AD_1_, adult in first year; AD_2_ etc., adult in year 2 etc.

Flowering plants of *O. flocktoniae* can be found all year, but peak flowering occurs from December to May. The inflorescence of *O. flocktoniae* is a capitulum of ∼80 flowers. The outer ray florets are white and ligulate (38·88 ± 1·09 flowers, *n* = 26) and the central tube-florets are yellow (38·32 ± 1·03 flowers, *n* = 26). In the first year of flowering, plants produce about six inflorescences (C. L. Gross, unpubl. data). Inflorescence production increases with age and is usually ∼100 (up to 400) inflorescences per plant on a 4-year-old plant. Inflorescences are visited by a wide variety of Lepidoptera and Hymenoptera (C. L. Gross, unpubl. data). The fruits are one-seeded achenes (3·5 mm long × 2 mm wide, with a pappus of numerous bristles, referred to as seeds from now on) and seed-to-flower ratios are high (∼80 %; C. L. Gross, unpubl. data) but not all seed are viable (see below). The breeding system varies in *O. flocktoniae*, as within populations some individuals do not self-pollinate and other plants can automatically self-pollinate (20–46 % fruit set; C. L. Gross, unpubl. data). Seed production in some years can be enhanced by pollen supplementations, whereby 40–60 % more fruits will be produced compared with control inflorescences that do not receive supplementary pollen (C. L. Gross, unpubl. data). *Olearia flocktoniae* is a diploid species (Flood, 2002) with *x* = 9 (Cross *et al.*, 2002). The life cycle of *O. flocktoniae* can be broken down into 12 stages (Fig. 1) using a biological stage classification approach combining reproductive and size criteria. We recognize two seedling stages, SD_1_ and SD_2_ (SD_1_ germinants, <15 d old, cryptic owing to their small size of 2 mm wide × 2 mm tall, with poor survivorship; SD_2_ seedlings, <20 cm tall, flowers/fruits absent); one juvenile stage that it not sexually reproductive (J_1_, >20 cm tall, flowers/fruits absent); and four stages for adults [AD_1_, first year flowering, about one or two flowering branches; AD_2_, 2-year-old plants, four or five flowering branches and 30 inflorescences; AD_3_, 3-year-old plants, six to ten branches and up to 200 inflorescences; and AD_4_, 4-year-old plants, ≥11 branches and 100 inflorescences (to 400 inflorescences)]. AD_5_ is an uncommon transition state for *O. flocktoniae* and we were unable to include it in the matrix modelling as we did not monitor the earlier transitions for these few plants. The seedling phase (SD_1_ + SD_2_) lasts about 3–12 months, the juvenile phase (J_1_) usually lasts up to 1 year (rarely 2 years), adults (flowering plants) occur from 12 months and in each succeeding year they rapidly increase in height and width by adding flowering branches. As a result of overcrowding from other species (especially *Ozothamnus diosmifolius* and *Gonocarpus oreophilus*) in year 4, large adult plants become smothered, and they then senesce and die.

### Demographic approach

The robustness of demographic data for subsequent life history analyses is likely to be sensitive to three key influences. These are: (1) variation in survival probabilities and fecundities among individuals (e.g. some seedlings may suffer greater mortality than others); (2) variation in the ecological conditions between census periods; and (3) covariation between the life cycle stages (e.g. individuals may be directly interacting to affect each other's vital rates). For SD_2_, J_1_ and the adult stages, we minimized these issues by (1) including all plants in the population in the census (2); sampling over many years; and (3) following cohorts throughout their lifetime via a longitudinal study. However, the presence of a seed bank and the cryptic SD_1_ first seedling stage (see above) meant that all survival probabilities and fecundities for *O. flocktoniae* could not be estimated from the same cohort. The SD_1_ and seed bank parameters were estimated in experiments (described below).

### Demography 1994–2013

Populations were located using forestry records, local informants and extensive field searching. A population was considered to be a discrete collection of individuals usually separated from another group of plants by 1000 m. Some groups of plants separated by at least 1000 m were treated as separate populations when landscape features segregated the groupings (e.g. dense forest plantations). Our census work was carried out in all known populations, giving a total of 133 populations over the 20 years. In any one year the highest number of populations known to us was 48. Populations ranged in size from 1 to 2514 plants and occupied ∼1–2500 m^2^. New populations were added to the census as they were discovered. A population was considered a *de novae* population if there were no previous records of the species at that location. Annual, systematic, demographic sampling of populations commenced in early 1994 and continued until the end of 2005 for all but two populations, where monitoring continued until 2013. In 2013 (year 20) all populations were revisited and a census was undertaken. Census work was conducted between March and July to capture flowering, fruiting and germination events. Detailed population maps showing the locations of individuals were maintained for all populations so that known plants could be revisited and their transition from seedling to adult recorded. To quantify the impact of disturbance on seedling density we assessed both during the census from 1998 to 2005. The presence of seedlings, the area they occupied and the level of disturbance around them was carefully noted as (a) no disturbance obvious, no obvious signs of leaf litter or soil disturbance, both layers intact with an unbroken soil surface (referred to as undisturbed sites henceforth), (b) moderate disturbance, recent evidence (<6 months old) of disturbance (e.g. disturbed leaf litter, plants slashed from road equipment) and (c) intensive disturbance, bare earth exposed recently (<6 months old, e.g. by roadside grading (grading is disruption to c. 1–20 cm of topsoil by a blade on a grader), lyrebird diggings).

### Estimating vital rates and matrix construction

#### Non-fertility matrix elements

The vital rates (i.e. coefficients of mortality and growth) were obtained from the fates of the plants in the census, i.e. the survivorship of a plant from SD_2_ to AD_4_. Over the 8 years we were able to track 98 cohorts over their lifetimes (*n* = 27 populations) starting from SD_2_. From the 98 matrices we calculated the range of lambda (λ, intrinsic rate of population growth). From this larger data set we selected ten populations (*n* = 3–6 populations per year) that waxed and waned between 1998 and 2005 (8 years). This gave 38 cohorts, of which four died in the year that they attained AD_1_, 11 died in the year that they attained AD_2_, 13 died in the year that they attained AD_3_ and ten died at AD_4_. This gave us 38 matrix models (see below) and a total of 190 vital rates replicating SD_2_ through to the adult stages.

Seedling stage SD_1_ is very difficult to document accurately in the field. It represents the cryptic germinants. On a number of occasions we found a dense flush of SD_1_ germinants (perhaps as a result of a whole seed head being interred into the soil) that would be short-lived, often due to scratching by birds in the leaf litter. We estimated the vital rates of SD_1_ from the seed bank experiment in the glasshouse (see below) and in the field at four populations.

#### Fertility matrix elements (fecundity and seed bank)

The vital rates for fecundity are transitions to seedlings from the adult plants or from the seed bank. We were unable to derive probabilities of flowering to seed emergence directly. Instead we derived estimates based on sampling inflorescence and seed production across many sites or from seed bank studies (see below). As adult plants grow they develop additional branches, which produce clusters of inflorescences. Seed production in the plants was estimated by multiplying the average number of seeds per infructescence by the average number of inflorescences produced on an adult each year. We estimate that in a year AD_1_ plants each produce ∼6 infructescences, AD_2_ plants 30 infructescences, AD_3_ plants 200 infructescences and AD_4_ plants, which are often senescing, ∼100 infructescences (sometimes 400 infructescences). An infructescence has the potential to produce ∼80 seeds (see above). Our glasshouse trials (see below) showed that seed viability is on average 58·4 % (fresh seed in 1996). In our model we applied this variable as a constant to the seed rain from adult plants.

*Olearia flocktoniae* has a soil seed bank, the abundance and viability of which we investigated using *in situ* field exhumations to look at seed bank longevity, *in situ* germination trials and *ex situ* glasshouse trials of germplasm longevity. We incorporated spatial and temporal variation in our experiments so as to set parameters for the vital rates to be used in the matrix models.

#### *In situ* seed bank longevity

We determined the abundance and longevity of seeds in the soil by using sites where plants were recently (1–5 years) extinct above ground and where the position of extirpated plants was identified by a marking peg placed adjacent to the plant when it was alive. In 1999, after annual survey data had been accrued for 5 years, a schedule of sites of known population ages of either extant or extinct above-ground populations was assembled. From the schedule of sites, 22 populations separated widely in the landscape were selected for a seed bank study based on the following parameters: ten were extant populations and 12 were extinct above-ground populations (extinct populations). The extant populations covered a broad geographical range and all had at least seven individuals extant at the time of selection (mean population size 45·20 ± 16·19, *n* = 12, range 7–144 adult plants). Five of the populations had been persistent at the same location for at least 5 years and the remaining five populations had only 1 year of fruiting in their history. The 12 extinct populations were mapped populations where plants were once above ground and included three populations that had each been extinct above ground for each of 5, 4, 2 or 1 year(s). Only sites with marker posts that indicated where a plant had once grown were used. Soil samples were collected from all 22 populations on 20 or 21 July 1999. One bottomless cake-tin (40 cm × 40 cm × 4 cm deep) was sunk into the soil at each of three positions in each site: (1) directly under a shrub, or adjacent to field markers that indicated where shrubs had once grown for the extinct populations; (2) 1 m away from the shrub/marker; and (3) 5 m from any *O. flocktoniae* plant or marker but within the pioneer habitat. Soil was scooped from tins and transferred to hessian bags (one bag per sampled position) and transported to the glasshouse, where soil was spread to a depth of 15 mm in trays over a vermiculite base and then placed under an automatic watering system for twice-daily saturation. Pilot studies have shown that *O. flocktoniae* grows readily from seed in the glasshouse without the need for dormancy-breaking treatments (C. L. Gross, unpubl. data). Temperatures in the glasshouse ranged from 7 to 35 °C.

#### *In situ* germination trials

Using fresh seed or viable seed from the germplasm collection (see below) we sowed seeds in the wild to determine natural germination rates. We made space in four populations by scraping a clearing to prepare a seed bed (total of 21 beds) and we sowed 25 seeds per seed bed, one seed per drill hole ∼2 mm deep. We lightly covered the seeds with local soil. We watered the seed bed and returned 2–4 weeks later to score germination.

#### *Ex situ* germplasm longevity

In 1996, five populations were selected for a seed longevity study. Populations were selected on the basis of age structure (adults present), reproductive state (fruiting in progress), population size (more than five individuals at any historical point) and a wide distance among sites. In March 1996, at least 500 seeds from at least five individuals from within each of the five populations were collected by hand, bulked within populations, counted into lots of 50 seeds and stored in envelopes in a dehumidifier (germplasm collection). In April 1996 (year 0) and every April for the following 9 years (1996–2005), one seed packet of 50 seeds from each site was randomly selected, and the 50 seeds were sown across a vermiculite tray in shallow rows (total of five trays). The five trays were then placed under a watering system in the glasshouse. Germination began within 10–12 d and germinability was scored at days 20 and ∼55. Ungerminated seeds were exhumed at the end of each trial and inspected for condition.

### Matrix construction

A linear time-invariant matrix population model after Caswell (1989) is given by

(1) $${\bf n}(t\hbox{} + \hbox{1})\hbox{} = \hbox{}A{\bf n}(t)$$

where the state vector **n(***t*) is a vector of *k* stage/age classes at time *t. A* is a Leslie matrix of *a_ij_* values that describe how each stage contributes to the number of individuals in all other stages at the next step. Life history parameters (fecundity, survivorship) were arranged into a Leslie matrix and analysed using PopTools version 3.2.5 (Hood, 2010) for λ (intrinsic rate of population growth, the absolute value of the dominant eigenvalue *A*), sensitivities and elasticities. The monitoring of the 27 populations over the 8 years gave us 98 matrices. From these we calculated 98 values for λ. We used a subset of 38 matrices from ten populations that had a range of population longevities (detected during the census; see above). We used standard deviation in PopTools to provide a confidence estimate for each eigenvalue. We produced an overall matrix by averaging vital rates (arithmetic means) from the 38 individual matrices (Supplementary Data Table S1). We compared sensitivity and elasticity analyses (proportional sensitivity) for our 38 matrices to determine whether the life-cycle stages most important to population growth varied among short- and longer-lived populations. The sensitivity values describe the relative response of *λ* to a perturbation. A high sensitivity in the transition between two life stages means that changes in this transition will have a strong effect on the population growth rate. The elasticity values are a proportional description of sensitivity, scaled to sum to 1, to allow easier comparison between the different stages.

### Additional statistical analyses

Linear regression was used to investigate the influence of population sizes on recruitment and the number of germinants, and separately for the relationship between population persistence and the number of disturbance events. To test for an effect of disturbance levels (undisturbed, moderate, intense) on seedling density, we dealt with the unbalanced design of site by year (not all 27 populations were extant continuously during 1998–2005), the unbalanced distribution of disturbance levels with the presence of seedlings (only one account of seedlings in an undisturbed area) and the potential for pseudo-replication of a site being used more than once in an analysis in the following way. We randomly sampled (1) ten unique populations across years from the dataset in which seedlings were growing in moderately disturbed sites and (2) ten different populations that had seedlings growing amid intense disturbance levels. We used one way ANOVA (log transformed) to test for an effect of disturbance on the number of seedlings per m^2^ as the dependent variable and disturbance level as the factor. As years became a random factor and site a unique factor, we omitted them from the model. We omitted from the analysis the single record of seedlings from the undisturbed site. Multifactor ANOVAs were used (1) to test for differences in germination against the factors of within-site position of soil cores and the number of years extinct; (2) to compare survivorship of seedlings, juveniles and adults; and (3) to compare λ against population longevity. A 1/square root transformation was required to homogenize variances for (1) and (3). Model I ANOVAs (fixed factors) were used in which the contribution of each factor was measured after having removed the effects of all other factors. Least significant difference *post hoc* tests were employed to dissect significant differences among treatment groups. Data were analysed using Statgraphics^®^ Centurion XV. Means and standard errors are presented as the central tendencies and dispersions except for the eigenvalues in the matrix, which had standard deviations.

## RESULTS

### Population census

**Fig. 2.**
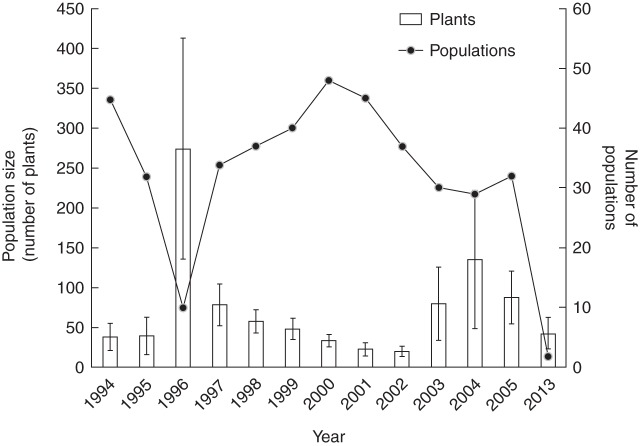
Number of extant populations and population size (mean number of plants ± s.e.) of *O. flocktoniae* from 1994 to 2013.

Over the 20 years, *O. flocktoniae* was found in 133 discrete populations, although in any one year far fewer populations (10–48) were extant above ground (Fig. 2). Population sizes (mean number of plants) varied significantly across years (*F*_11,405_ = 3·08, *P* < 0·001; Fig. 2). In populations where both colonization and extirpation were recorded, the average number of years that a population persisted above ground was 2·80 ± 0·29 (s.e.) (range 1–6 years, *n* = 27). However, there were some populations (9 %) that were established before this study (pre-1994) and were persistent for at least 7 years, three populations were extant for 12 years and two persisted for the 20 years of the study. Only 9 % of populations were established as primary colonization events in new localities during this study. All other ‘new’ populations re-established at known, previously colonized sites.

**Fig. 3.**
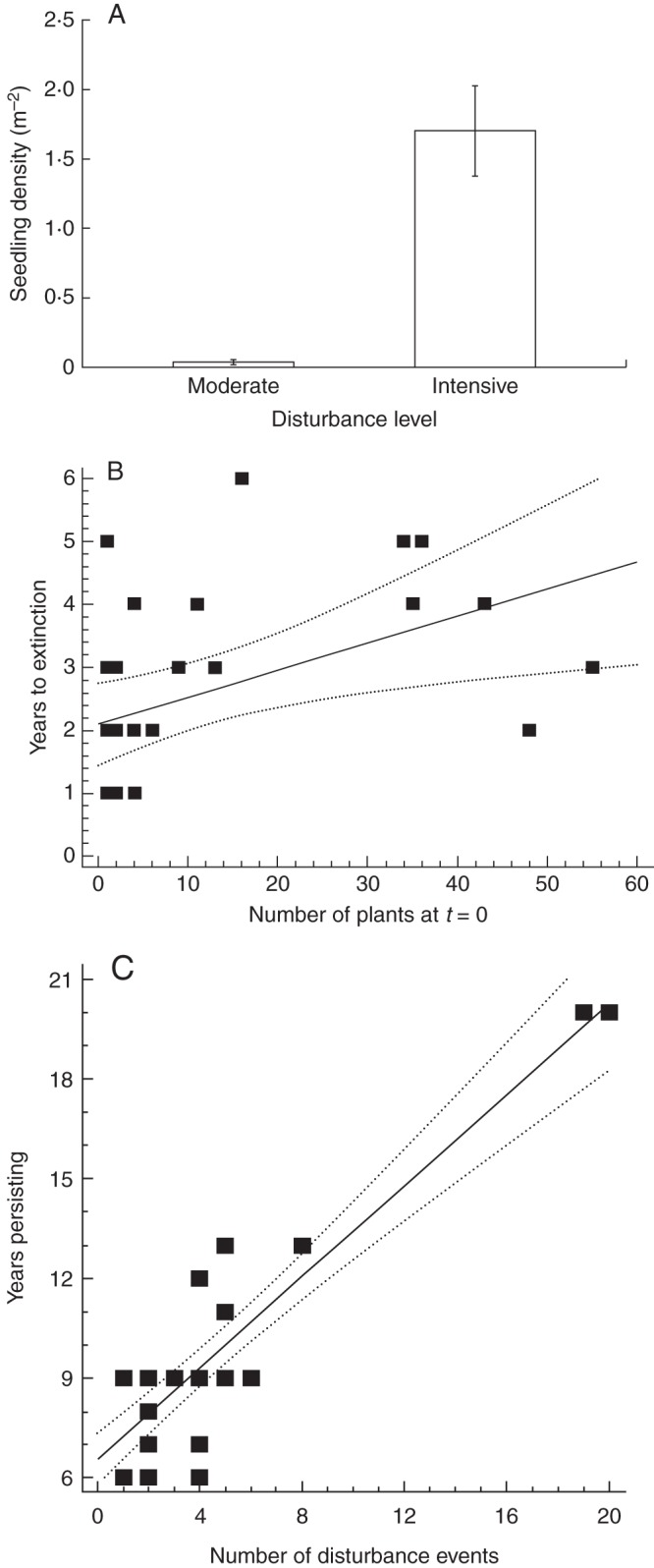
(A) Relationship between disturbance levels (moderate and intense) and the density of seedlings per m^2^ ± s.e. Moderate disturbance is recent evidence (<6 months old) of disturbance (e.g. disturbed leaf litter, plants slashed by road equipment); intensive disturbance is bare earth exposed recently (<6 months old, e.g. by roadside grading, lyrebird scratchings). The differences were significant (*F*_1,18_ = 146·18, *P* < 0·0001). A third class of disturbance, undisturbed (see the Methods section for details) was only associated with seedlings once, with five seedlings over 25 m^2^. (B) Relationship between the number of plants that establish at time = 0 and the longevity of the population (years to extinction) (*R*^2^ = 0·22, *P* = 0·01). (C) Relationship between disturbance and population persistence (number of disturbance events) (*R*^2^ = 0·83, *P*<0·0001).

Most populations (76 %) went extinct above ground at least once during the 12 years and in 18 of these populations plants re-established from the seed bank at the same location 1–4 years later (1·88 ± 0·25 years absent, *n* = 18). The number of seedlings recorded in the first year post-recovery was variable (24·27 ± 7·64, range 1–127) and not related to the size of the population in the last year (*R*^2^ = 0·0011, *P* = 0·89), but was strongly influenced by disturbance levels in the previous 6 months, with intensively disturbed sites having significantly more seedlings per m^2^ than moderately disturbed areas with seedlings (*F*_1,18_ = 146·18, *P* < 0·0001; Fig. 3A). Undisturbed areas with seedlings were only detected once (five seedlings over 25 m^2^).

Over the 20 years, 27 populations were noted where both colonization and then extinction occurred (at new and established locations). In these situations it was found that the number of plants to establish at *t* = 0 had a significant but moderately weak and positive influence on the longevity of the population (years to extinction, *R*^2^ = 0·22, *P* = 0·01; Fig. 3B). The length of time that a population persisted at a site was directly influenced by the number of disturbance events (road grading): the more disturbance a population received, the longer a population persisted (*R*^2^ = 0·83, *P* < 0·0001; Fig. 3C) and intense disturbance was associated with higher seedling density than moderate or undisturbed situations (Fig. 3A).

### *In situ* seed bank longevity

**Fig. 4.**
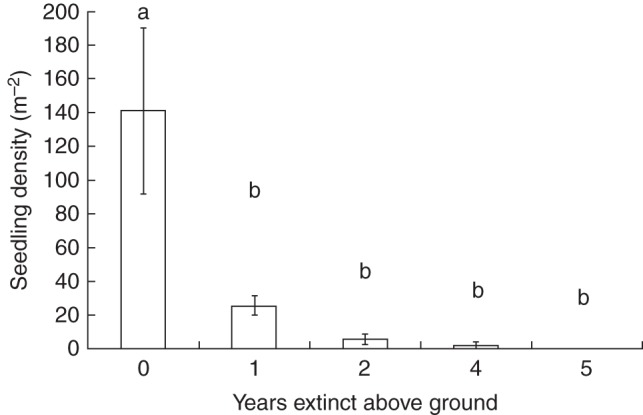
Seedling density (mean number of seedlings/m^2^ ± s.e.) from soil cores taken from sites (within-site positions averaged) where *O. flocktoniae* was extinct above ground for 5, 4, 2 or 1 year/s and extant above ground (year = 0). Bars that do not share the same letter are significantly different at *P* < 0·05.

Seedlings emerged from soil cores retrieved from all sites except the three sites where the species had been extinct above ground for 5 years (Fig. 4). ANOVA showed that there was a significant difference in the number of germinants recoverable from the different extinction treatments but that there was not a significant effect of within-site location of soil cores or a significant interaction between age of extinction and soil core position (Table 1). Of the 725 seedlings to emerge from the 66 seed cores, ∼93 % of were derived from the soil from extant populations (Fig. 4). Within this group, more seedlings were recorded from soil cores taken from populations extant for 5 years compared with those extant for 1 year but the difference was not significant (5 years old, 171·67 ± 54·76 seeds/m^2^; 1 year old, 110·42 ± 86·19 seeds/m^2^; *F*_1,8_ = 0·36, *P* = 0·56), with a pooled average of 141·04 ± 49·21 seeds/m^2^ (Fig. 4). *Post hoc* tests revealed that the number of germinants from extant populations was significantly greater than those from populations extinct for 1, 2, 4 and 5 years (Fig. 4). Recovering plants from seed cores is thus more likely from extant than from extinct sites.

**Table 1.** Multifactor ANOVA of number of germinating seedlings from seed core experiments

Source	Sum of squares	d.f.	Mean square	*F* ratio	*P* value
Main effects					
Years extinct	5·21	4	1·30	5·94	0·0005
Core position	0·18	2	0·07	0·31	0·7321
Interaction					
Years extinct × core position	0·48	8	0·06	0·27	0·9721
Residual	11·83	51	0·22		
Total (corrected)	17·19	65			

Core position refers to the three positions in each site from which soil cores were excavated (see text).

### *In situ* germination trials

None of the seeds sown in the wild populations germinated in any year (21 seed beds across four populations, a total of 525 seeds). Seeds from the same batch as that used in greenhouse trials (see *ex-situ* germplasm longevity trials above and below or in 2010 as part of unpublished work) exhibited moderate levels of germinability (>40 %), indicating that a lack of viability was not the only reason for poor field germination.

### *Ex situ* germplasm longevity

**Fig. 5.**
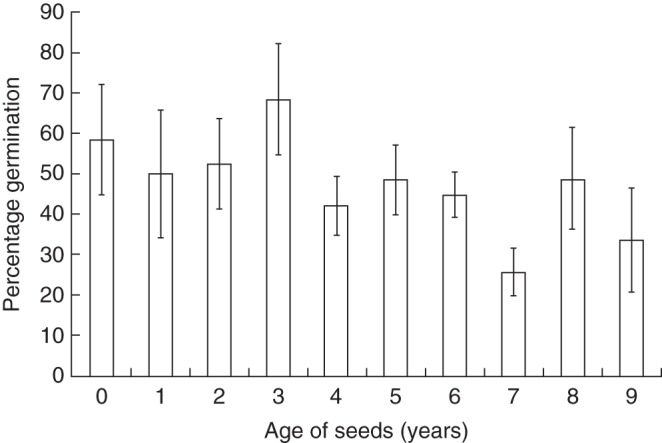
Percentage germination (mean± s.e.) at day 55 for seeds collected in 1996 and tested annually for germinability in the glasshouse in years 1996–2005. *P* = 0·63, not significant.

The 10-year trial involving seeds collected in 1996 from five sites showed that germinability did not vary significantly over time (1996–2005, *F*_9,49_ = 0·79, *P* = 0·63; Fig. 5). The average germinability across years by day 20 was 40·32 ± 3·05 % (range 0–82 %). At the point when no new seeds had germinated (day ∼55) there was still no difference in germinability among years (*F*_9,49_ = 1·14, *P* = 0·36) and here the average germinability across years was 47·24 ± 3·60 % (range 0–96 %). Exhumations of un-germinated seeds from trays at the end of the trials in all years showed that these seeds had decomposed.

### Life history: survivorship and fecundity

**Fig. 6.**
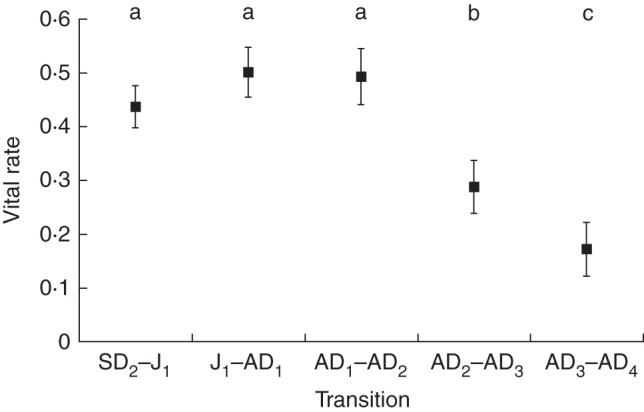
Vital rates (mean ± s.e.) for five transitions from seedlings (SD_2_), juveniles (J_1_) and adults (AD) aged 1–4 years. Points that do not share the same letter are significantly different at *P* < 0·05.

**Fig. 7.**
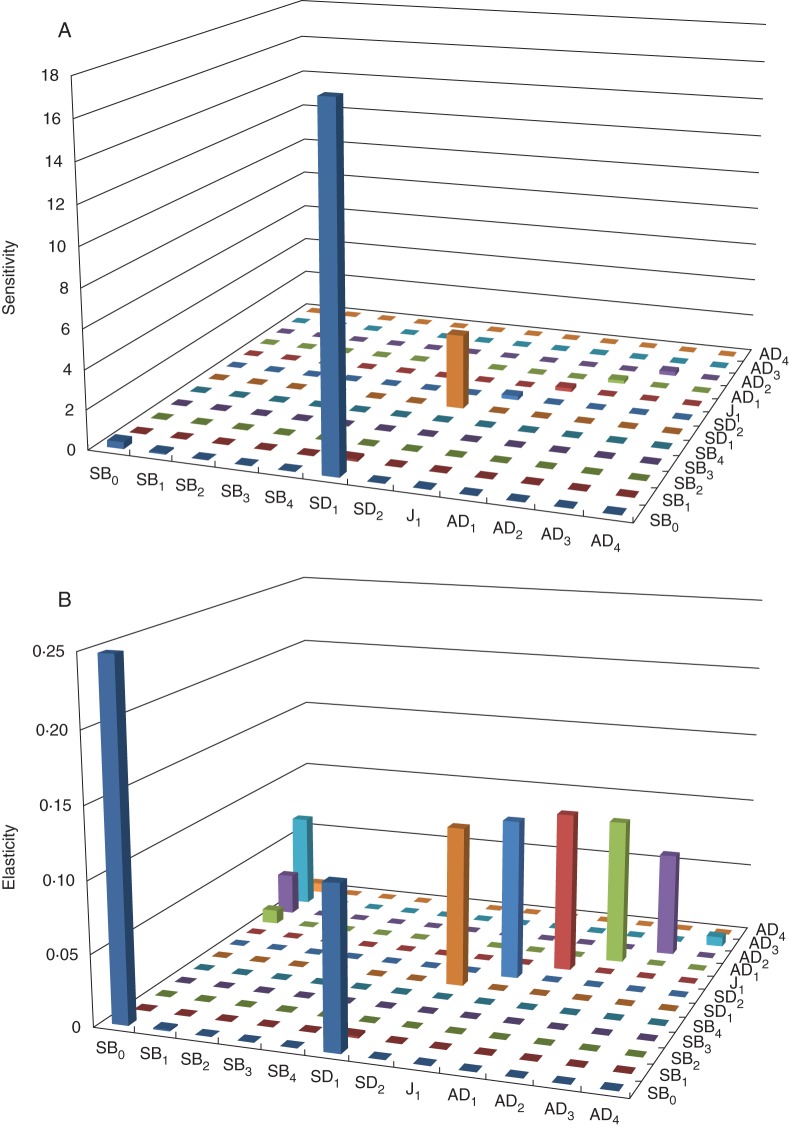
Sensitivity matrix (A) and elasticity matrix (B) for *Olearia flocktoniae* based on averages of vital rates from 38 matrices and ten populations.

We used 27 populations (98 cohorts) to look at variation in λ and ten populations (38 cohorts, 190 transitions) to look at vital rates in detail. The λ value was found to range from 0·58 to 1·10 and was only ever >1 in five cohorts. Vital rates varied from SD_1_ to AD_4_ (Fig. 6 and Supplementary Data Table S1). A matrix based on the average of 38 cohorts is given in Supplementary Data Table S2. Using sensitivity analyses on the vital rates for the 12 life history stages for *O. flocktoniae*, we found the fate of SD_1_, which is affected by many factors, had on the whole the greatest influence on the persistence of populations (Fig. 7A). Elasticity analysis showed that a 1 % increase in depositing seeds into the seed bank will cause an increase in λ of 0·25 (Fig. 7B). Sensitivities and elasticities did not vary within stages and between the final adult stages reached in a population (i.e. AD_1_, AD_2_, AD_3_ or AD_4_), although among stages the differences were significant, as expected (Fig. 7A, B). From a potential seed rain of 22 848 viable seeds (in a population that has at least one plant in each adult age class, AD_1_ to AD_4_ plants), <0·9 % of seeds survived to germinate during the 4 years after dispersal. The majority of this 0·9 % of seed will germinate in the first year (93·38 %), 5 % in the second year, 1·09 % in the third year and 0·42 % in the last year. Only 2·5 % of SD_1_ survive to become older seedlings. Once plants are at this second seedling stage (SD_2_), survivorship improves, with 44 % of seedlings surviving to become juveniles (J_1_), 50 % of juveniles surviving to become adults (AD_1_) and 49 % of adults surviving to their second year. Thereafter, survivorship decreases to 29 % for plants transitioning to their third year and only 17 % of these plants survive to their fourth year (Supplementary Data Table S2).

## DISCUSSION

Ecological studies of species passively translocated to new areas have mainly focused on alien plant species, which has yielded much information on the life history parameters influential on survival and reproduction [e.g. *Cytisus scoparius* (Parker, 2000; Simpson *et al*., 2005); *Phyla canescens* (Gross *et al*., 2010)], including the findings that high levels of seed production and seed bank persistence are two key determinants of population persistence but that dominance can shift with a change in environmental conditions [*P. canescens* (Price *et al*., 2010, 2011)]. The key difference from passively translocated *O. flocktoniae* is that the seed banks are transient. Passively translocated rare species appear to be uncommon, or at least poorly studied, compared with more sedentary and iconic threatened species [e.g. *Macadamia* spp. (Pisanu *et al*., 2009; Neal *et al*., 2010); *Tetratheca juncea* (Gross *et al*., 2003)] or rapid-colonizing cosmopolitan species [*Canavalia rosea* (Gross, 1993)]. However, by studying these different life history patterns (rare and common, native and alien) we can learn much about the drivers and consequences of passive translocation, which is likely to be a more common scenario under changing climatic conditions. *Olearia flocktoniae* is a rare species, yet it is an early pioneer with characteristics of many weedy species (e.g. large numbers of seeds produced, transient seed bank). We established that it is prone to transiency and local extinction. Re-establishment at sites is influenced by the time elapsed since extirpation, with 4 years as a maximum period for successful re-appearance. The number of seedlings that re-establish at sites is also influenced by the total number of plants that existed over all years in the population prior to extinction. The marked difference in longevity between seeds in seed banks and *ex situ* seed collections indicates that the seeds of *O. flocktoniae* rapidly decline in vigour once in the seed bank or are lost to the population through predation. This is supported by the few plants that re-establish in revived sites despite the production of many tens of thousands of seeds in the year prior to extinction. Furthermore, none of the field-sown seeds germinated, despite being viable. In the greenhouse, ungerminated seeds were excavated from trays where it was found that they had rotted, suggesting that decay is a major constraint on *in situ* seed longevity. Transient seed banks are usually considered to be those that germinate within a year of seed banking (for a discussion see Walck *et al.*, 2005) and ‘persistence’ is a broad catch-term for anything that persists past a year. Trials showed that the seed bank of *O. flocktoniae* is functionally short-term-persistent in the field but potentially long-term-persistent. However, ‘transient’ is an appropriate descriptor of the seed bank for *O. flocktoniae* as ∼93 % of seeds that will ever germinate do so in the first year. The key vulnerabilities in the life cycle of *O. flocktoniae* are that most seeds never germinate in the field and those that do rarely survive to the robust seedling stage (SD_2_). Seed survival has been highlighted as a crucial stage in life history analyses of both endangered [e.g. *Helenium virginicum* (Adams *et al.*, 2005)] and invasive [e.g. *Ambrosia artemisiifolia* (Fumanal *et al.*, 2008)] Asteraceae. Furthermore, Alvarez-Aquino *et al.* (2005) found that human activities influenced the size and composition of seed banks in disturbed cloud-forest fragments, the least-disturbed habitats containing a greater proportion of species with dormant seeds. Thus, human-induced disturbance, as seen in *O. flocktoniae* populations, may replace the natural disturbance events previously found in the species' natural range that removed competitors and stimulated germination of transient seed banks. There are some 180 species of *Olearia* within Australia, New Zealand and New Guinea (Lander, 1992) and many are pioneer species that occupy successional sequences in dynamic landscapes [e.g. *Olearia argophylla* (Ashton, 2000)].

The seed bank of ∼141 seeds/m^2^ in *O. flocktoniae* is low relative to its seed rain or compared with invasive Asteraceae [e.g. *Ambrosia artemisiifolia*, 536–4477 seeds/m^2^ (Fumanal *et al.*, 2008)]. It is, however, larger than that found for other Australian plant communities (see Fig. 2 in Vlahos and Bell, 1986; Gross and Vary, 2014), including seed bank studies from the adjacent moist New England Tablelands [e.g. 18·5 seeds/m^2^ for the native Asteraceae *Brachyscome aculeata* (Table 1 in Grant and MacGregor, 2001); <13 seeds/m^2^ for four hard-seeded species (Campbell and Clarke 2006)]. Clearly, seed bank densities are quite variable and 141 seeds/m^2^ in itself is not the major problem with population persistence in *O. flocktoniae* [see e.g. Table 3 in Crawford and Young (1998), where the majority of 25 species have seed bank densities <5 seeds/m^2^].

Survival of SD_1_ was very low in this study and this is the most vulnerable stage of the life cycle. Only 2·5 % of SD_1_ survived to the SD_2_ seedling stage, which sensitivity and elasticity analyses showed to be key vulnerabilities. Natural processes [e.g. the subsequent digging of disturbed areas by superb lyrebirds, *Menura novaehollandiae* (C. L. Gross, pers. obs.)] contribute to poor survivorship at this stage. The SD_1_ stage comprises seedlings that are very easily overlooked in the field. This important phase of seedling establishment has the potential to skew the analysis of life histories if overlooked. This may account for unexplained gaps in establishment detected in other systems where there were uncertainties in seedling emergence and establishment (e.g. Dalling *et al.*, 1998).

Seedlings, juvenile and adult phases of *O. flocktoniae* were the most robust stages in the life cycle of this species. More than 44 % of SD_2_ seedlings survived to become juveniles, 50 % of juveniles survived to adulthood and 49 % of 1-year-old adults survived to flower another year. Two of the study populations persisted for the 20-year duration of this study. Their habitats were maintained as open simple communities. This occurred through careful site maintenance (removal of competing vegetation and soil disturbance) and signs that informed road workers of plant positions.

### Primary colonization versus secondary colonization

During the 12 years of the study, much of the forest network of roads and new disturbance events (e.g. logging activities) were traversed and examined for recruitment. Primary colonization occurred in 9 % of populations. These were sites where there were no plants present prior to these disturbance events (as evident from our census results coupled with seed bank longevity projections). The seed vector for these colonization events was most likely heavy machinery (e.g. bulldozers), which traverses the roads or is moved between forest patches on trucks. Seedlings in primary populations were always clumped and the mean population size at *t* = 0 was 14·67 ± 4·17 (*n* = 12 populations). Primary colonization events in other habitats in the forest interior may have occurred and been missed during annual surveys. However, the potential for long-distance dispersal is likely to be poor in this species because of low wind in these managed plantations, which are densely planted. Wet conditions further contributed to poor dispersal as it was noted that many soggy infructescences failed to disperse their seeds (C. L. Gross, unpubl. data). Opportunities for wind dispersal to interior patches are unlikely from the wet margins of these rainforest environments. Strykstra *et al.* (1998) found that the rare daisy *Arnica montana* was inefficient at long distance dispersal in the Dutch landscape. In wind-tunnel experiments they found that only poor quality achenes (the lightest achenes, which also had poor germination capabilities) could disperse long distances and they remarked that humans were the probable vectors for long distance dispersal (Strykstra *et al.*, 1998).

The displacement of *O. flocktoniae* to new sites has not been a deliberate action of conservation translocations, but there are similarities in species' response. *Olearia flocktoniae* has an inadequate seed bank for population persistence, an obstacle that has hampered translocation efforts in other endangered species [e.g. *Holocarpha macradenia*, (Holl and Hayes, 2006)] and has been the impetus for recommending the translocation of seedlings rather than seeds (Jusaitis *et al.*, 2004). *Olearia flocktoniae* is currently conservation-dependent on annual disturbance at extant wet sites, but it would be worth trialling translocation to drier sites. Sites with low soil water retention may be the best option for population persistence of *O. flocktoniae* in a landscape. Low levels of moisture, low light and low levels of fluctuating temperatures have been attributed to seed bank persistence in other pioneer systems (Tsuyuzaki and Goto, 2001). In *Chromolaena odorata* (Asteraceae), Witkowski and Wilson (2001) found that the provision of less water significantly improved seed persistence in the soil for this invasive species. Arroyo *et al.* (2006) studied annual and perennial herbs of *Chaetanthera* (Asteraceae) and found that a persistent seed bank was correlated with aridity in the perennial and annual species, with species from more arid areas having larger persistent seed banks.

### Long-term conservation implications

*Olearia flocktoniae* is currently conservation-dependent as the only habitat of persistent populations currently available is artificially created by humans. The nature and location of natural habitats of *O. flocktoniae* in the landscape can only be hypothesized now. A likely scenario is that *O. flocktoniae* existed on the scree slopes of the east-facing Great Dividing Range near Dorrigo. Many of these habitats are now occupied by invasive *Lantana camara* and small-leaved privet (*Ligustrum sinense*). *Olearia flocktoniae* requires sites where natural disturbance events provide an open habitat for regeneration, a feature required in other species of *Olearia* [e.g. *O. adenocarpa* (Heenan and Molloy, 2004); *O. axillaris* (Hinchliffe and Conran, 2005); *O. polita* (Williams and Courtney, 1995) and *O. heterocarpa*, which exists in a boom-and-bust cycle with natural landslips on the obsidian scree slopes of the Mt Warning caldera, 220 km north of Dorrigo]. In other studies, fire provided the appropriate disturbance for regenerating populations [e.g. *Dicerandra frutescens* (Menges *et al.*, 2006)] and is worth trialling for *O. flocktoniae* as a technique to open up habitats (fire is not needed for germination, but seedlings do colonize burnt log dumps), although it may be difficult to employ in these forests managed for timber production. In addition, fire as a management tool may have unexpected and negative consequences for the breeding structures of populations (Gross *et al.*, 2012) when the frequency and intensity are unnatural.

In a study of three species of Asteraceae, Eriksson (1997) found support for the hypothesis that the abundance of a species reflects its colonizing ability. Results from *O. flocktoniae*, a species in low abundance in the landscape, also support this hypothesis as no colonization events occurred that appeared to be derived from natural long-distance dispersal, i.e. poor species abundance reflects poor colonization ability.

### Displacement ecology: the impact of a habitat shift on survivorship

The survivorship of displaced organisms (e.g. as facilitated by humans or under climate change scenarios) is of fundamental importance to ecosystem continuity, species persistence and the evolutionary longevity of species. This study showed that seed bank survivorship and SD_1_ survivorship were the most vulnerable phases in the life cycle of *O. flocktoniae*, a displaced species.

SD_1_ plants are very vulnerable to the arrays of new predators and other threats that may be encountered in displaced environments. The superb lyrebird, a native forest floor forager, was recorded digging up and killing *O. flocktoniae* SD_1_ in this study during its normal foraging activities. These foragers are specialists in humid forests and play a significant role in shaping the habitats of other species (Webb and Whiting, 2006). In a drier habitat, such as the exposed slopes of the Great Dividing Range, *O. flocktoniae* may not have encountered them. Exposure to new soil pathogens, such as saprophytic fungi (Wagner and Mitschunas, 2008), may also be a threat to seed banks in habitats with altered climate regimes (see also Leishman *et al.*, 2000).

The long-term health of the seed bank has long been linked to environmental parameters such as humidity and temperature (p. 81 in Fenner and Thompson, 2005). The relative magnitudes of the effects of altered water regimes on seeds, however, has not been fully appreciated (p. 134 in Fenner and Thompson, 2005) and extrapolation to climate change consequences for seed banks requires further studies to match the important efforts on the perturbations to other plant phenological (Bertin, 2008; Hughes, 2000) and physiological (Betts *et al.*, 2000; Lloyd and Farquhar, 2008) events. We suggest here that studies should focus on the effects of altered rainfall regimes on community-level responses in the persistence and germination of seed banks. Population models will be particularly useful for this work (e.g. Levine *et al.*, 2008). In Australia and elsewhere, these approaches will be informative for areas that are experiencing increased rainfall events (more frequent events and a greater volume of rain) as a result of climate change [e.g. northwest Australia (Nicholls, 2006)]. This is important because the integrity of the covert populations will shape the future of overt populations.

### Conclusions

Demographic census is a powerful tool for detecting and diagnosing decline (Keith, 2002; Menges, 1990). The application of life history analyses (e.g. Gross, 2005) to pioneer species is shown here to be a powerful tool for evaluating population persistence, especially when there is temporal and spatial replication of population extinctions across the landscape. The low abundance of *O. flocktoniae* over the landscape reflects its poor colonizing ability—no populations were detected where unassisted dispersal was likely. Poor *in situ* longevity of the seed bank and an absence of a robust SD_1_ stage are the key parameters that undermine population persistence in this species and necessitate active management of the species until environmental conditions permit sustainable establishment. Studying the ecology of displaced species, especially the responses of their seed bank to disturbance, is important for understanding and predicting the consequences of climate change. This has implications for many species that need to relocate away from environments that have become hostile as a consequence of climate change, particularly when suitable environments have not yet become available.

## SUPPLEMENTARY DATA

Supplementary data are available online at www.aob.oxfordjournals.org and consist of the following. Table S1: summary of vital rates for the transitions and lambda from 1998–2005 in 38 cohorts. Table S2: the average matrix (*n* = 38) from 10 populations.

Supplementary Data
