# Supplementary material for: Two decades of demography reveals that seed and seedling transitions limit population persistence in a translocated shrub
Source: Ann Bot. 2014 May 20;114(1):85–96. doi: 10.1093/aob/mcu082 (PMC4071100; doi:10.1093/aob/mcu082)
Supplement: Supplementary Data [file supp_114_1_85__index.html]

Two decades of demography reveals that seed and seedling transitions limit population persistence in a translocated shrub — Two decades of demography reveals that seed and seedling transitions limit population persistence in a translocated shrub — Supplementary Data 

# Two decades of demography reveals that seed and seedling transitions limit population persistence in a translocated shrub

## Supplementary Data

Supplementary Data

**Files in this Data Supplement:**

- Supplementary Data - Pdf file
