# Supplementary material for: Two decades of demography reveals that seed and seedling transitions limit population persistence in a translocated shrub
Source: Ann Bot. 2014 May 20;114(1):85–96. doi: 10.1093/aob/mcu082 (PMC4071100; doi:10.1093/aob/mcu082)
Supplement: Supplementary Data [file supp_mcu082_mcu082supp_data.pdf]

SUPPLEMENTARY DATA

Table S1. Summary of vital rates for the transitions and lambda from 1998–2005 in 38 cohorts. See text for defintion of transition states. Later transitions are often 0 as plants did not live beyond year 3. Data are means ± s.e. (s.d.) Lambda was also calculated using the larger data-set of 98 matrices and was found overall to be 0.76 ± 0.01 (0.13).

Year	No. of cohorts	SD_2 to J	J_1 to AD_1	AD_1 to AD_2	AD_2 to AD_3	AD_3 to AD_4	Lambda
1998	6	0.43±0.08(0.19)	0.51±0.10(0.25)	0.64±0.13(0.31)	0.10±0.10(0.24)	0.00±0.00(0.00)	0.81±0.005(0.06)
1999	6	0.58±0.08(0.20)	0.40±0.12(0.28)	0.38±0.12(0.29)	0.42±0.09(0.21)	0.00±0.00(0.00)	0.76±0.01(0.13)
2000	6	0.45±0.10(0.24)	0.29±0.09(0.23)	0.37±0.12(0.30)	0.33±0.04(0.10)	0.51±0.13(0.31)	0.69±0.01(0.11)
2001	4	0.21±0.10(0.19)	0.32±0.11(0.22)	0.42±0.20(0.39)	0.11±0.04(0.08)	0.33±0.23(0.45)	0.76±0.02(0.15)
2002	3	0.60±0.20(0.35)	0.50±0.29(0.5)	0.75±0.16(0.28)	0.78±0.15(0.25)	0.25±0.25(0.43)	0.87±0.03(0.22)
2003	4	0.53±0.12(0.23)	0.73±0.12(0.23)	0.16±0.16(0.31)	0.38±0.24(0.48)	0.10±0.10(0.20)	0.71±0.01(0.08)
2004	5	0.23±0.09(0.19)	0.74±0.06(0.12)	0.74±0.12(0.27)	0.07±0.07(0.15)	0.20±0.20(0.44)	0.75±0.01(0.09)
2005	4	0.49±0.09(0.17)	0.61±0.09(0.17)	0.54±0.05(0.09)	0.30±0.20(0.35)	0.00±0.00(0.00)	0.89±0.01(0.08)
All years	38	0.43±0.038(0.24)	0.50+0.045(0.28)	0.49±0.05(0.32)	0.29±0.05(0.30)	0.17±0.05(0.31)	0.77±0.01(0.13)

Table S2. The average matrix (n = 38) from 10 populations. SB_0 = seed bank in the first year at time 0, SB_1 = seed bank in the second year at time 1, etc.; SD1 = germinants <30 d old, SD_2 = seedling >30 d old, <20 cm tall, J_1 = vegetative juvenile, AD_1 = adult in first year, AD_2 = adult in year 2, etc..

	SB_0	SB_1	SB_2	SB_3	SB_4	SD_1	SD_2	J_1	AD_1	AD_2	AD_3	AD_4
SB_0	0.58	0	0	0	0	0	0	0	408	2040	13600	6800
SB_1	0.0056	0	0	0	0	0	0	0	0	0	0	0
SB_2	0	0.0023	0	0	0	0	0	0	0	0	0	0
SB_3	0	0	0.0018	0	0	0	0	0	0	0	0	0
SB_4	0	0	0	0.0014	0	0	0	0	0	0	0	0
SD_1	0.0054	0.0039	0.0012	0.0011	0.0013	0	0	0	0	0	0	0
SD_2	0	0	0	0	0	0.025	0	0	0	0	0	0
J_1	0	0	0	0	0	0	0.436906	0	0	0	0	0
AD_1	0	0	0	0	0	0	0	0.501418	0	0	0	0
AD_2	0	0	0	0	0	0	0	0	0.493137	0	0	0
AD_3	0	0	0	0	0	0	0	0	0	0.288666	0	0
